# Synthesis and Structural Characterization of the New Clathrates K_8_Cd_4_Ge_42_, Rb_8_Cd_4_Ge_42_, and Cs_8_Cd_4_Ge_42_

**DOI:** 10.3390/ma9040236

**Published:** 2016-03-25

**Authors:** Marion C. Schäfer, Svilen Bobev

**Affiliations:** Department of Chemistry and Biochemistry, University of Delaware, Newark, DE 19716, USA; schaefer@udel.edu

**Keywords:** clathrates, type-I structure, germanium, cadmium, Zintl phases

## Abstract

This paper presents results from our exploratory work in the systems K-Cd-Ge, Rb-Cd-Ge, and Cs-Cd-Ge, which yielded the novel type-I clathrates with refined compositions K_8_Cd_3.77(7)_Ge_42.23_, Rb_8_Cd_3.65(7)_Ge_42.35_, and Cs_7.80(1)_Cd_3.65(6)_Ge_42.35_. The three compounds represent rare examples of clathrates of germanium with the alkali metals, where a d^10^ element substitutes a group 14 element. The three structures, established by single-crystal X-ray diffraction, indicate that the framework-building Ge atoms are randomly substituted by Cd atoms on only one of the three possible crystallographic sites. This and several other details of the crystal chemistry are elaborated.

## 1. Introduction

Clathrates of silicon and germanium with the alkali metals have been known now for almost half a century [1]. The idea of phonon-glass electron-crystal (PGEC), coined by G. Slack [2], succinctly captures the two most important structural features of these clathrates: rigid open frameworks, made up of covalently bonded atoms, and guest atoms residing in the large cages. The stable frameworks contribute to the favorable carrier mobilities, while the filler atoms that vibrate (or “rattle”, as is frequently said) in the oversized cages are believed to be the reason for the low lattice thermal conductivity exhibited by many clathrates. Therefore, this class of compounds has gained interest for their prospects as thermoelectric materials [2,3,4,5].

Most studied clathrate compounds are based on the group 14 elements Si, Ge, and Sn (*Tt*). These atoms form extended networks adopting several different structure types. Fractions of the framework atoms can be substituted by group 13 and 12 elements, as well as late transition metals (*M*), which can be stabilized in tetrahedral coordination. There are large cages in the clathrate structures, which are partially or fully occupied by guest atoms (*A*). The typical guest atoms are alkali metals, alkaline earth metals, or Ce and Eu from the rare earth metals [6,7,8,9,10].

**Figure 1:**
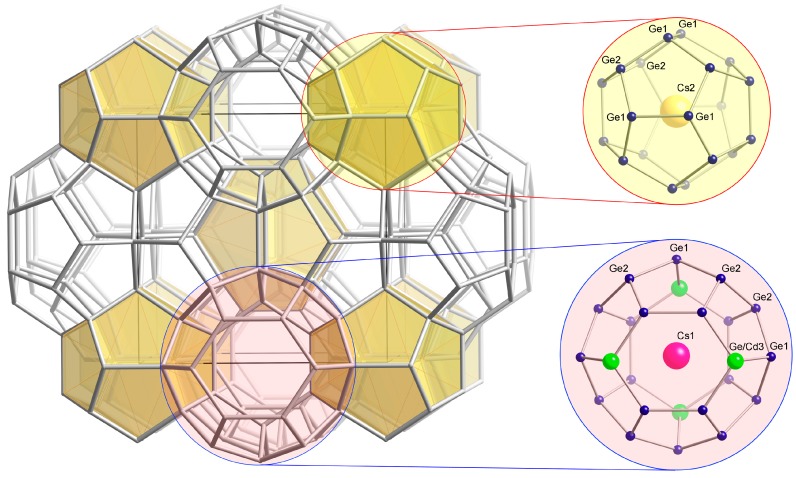
Schematic view of the clathrate with type-I structure. For the drawing, the structural information for Cs_7.80(1)_Cd_3.65(6)_Ge_42.35_ was used and the atoms are labeled accordingly.

This paper deals with three new clathrate phases with compositions K_8_Cd_3.77(7)_Ge_42.23_, Rb_8_Cd_3.65(7)_Ge_42.35_, and Cs_7.80(1)_Cd_3.65(6)_Ge_42.35_, which adopt the type-I structure (Figure 1). The nominal formula for this structure is *A*_8_(Cd,Ge)_46_, and it boasts cages of 20- and 24-atoms. Here, we would like to mention that numerous clathrates of germanium with the alkali metals are known as of today, but this study is among very few to identify cadmium as a substituent of germanium-based clathrates—the quaternary phase K_6_Eu_2_Cd_5_Ge_41_ appears to be the only report of similar chemistry so far [11]. The corresponding systems K-*M*-Ge and Rb-*M*-Ge with *M* being the group 12 elements Zn and Hg have already been explored, and the structures of the type-I clathrates K_8_Zn_4_Ge_42_ [12], K_8_Hg_3.19_Ge_42.81_, and Rb_8_Hg_3.03_Ge_42.97_ [13] have been published.

## 2. Results

### 2.1. Crystallographic Analysis

The structure solutions and refinements proceeded in a straightforward manner (Table 1), and only a few details deserve special mention. First, the contrast between the X-ray atomic scattering factors of Cd and Ge is significant, allowing precise refinements of the site occupation factors should there be mixed-occupied sites. Indeed, in all cases, when the occupancies of the framework sites were first freely refined (individually, while the remaining ones were kept fixed), it was noted that the occupation factors for 16*i* and 24*k* sites did not deviate from 100%, while sites 6*c* in all three structures appeared to be “heavier”. This indicates that Cd (*Z* = 48) and Ge (*Z* = 32) co-occupy framework site 6*c* only (in a ratio *ca.* 60% Cd, 40% Ge). Such distribution of group 12 and group 14 elements is consistent with the published refinements for K_8_Hg_3.19_Ge_42.81_ and Rb_8_Hg_3.03_Ge_42.97_ [13], K_8_Zn_3.46_Si_42.54_ and Rb_7.86_Zn_3.63_Si_42.37_ [14], and K_8_Zn_3.78_Sn_42.22_, Rb_8_Zn_3.52_Sn_42.48_, and Cs_8_Zn_3.44_Sn_42.56_ [15]. If one considers the alkaline earth clathrates, such as Ba_8_Zn_7_Si_39_ [16], the 6*c* site is also mostly occupied by Zn (77% Zn, 23% Si), with the remainder of the Zn found at 24*k* (9% Zn, 91% Si). 

Notice that, in all three structures, the Zintl-Klemm concept [17], which appears to be followed by many other clathrates [8], is not completely satisfied by the above-mentioned compounds. For instance, based on the rationalization [*A*^+^]_8_[*M*^2−^]_4_[*Tt*^0^]_42_, one should expect the structural formulae to be *A*_8_*M*_4_*Tt*_42_ (*A* = alkali metal, *M* = Zn, Cd, Hg; *Tt* = tetrel, or Si, Ge, Sn). The same holds true for Ba_8_Zn_8_Si_38_ (=[Ba^2+^]_8_[Zn^2−^]_8_[Si^0^]_38_). The reasons for the slightly lower refined content of Zn, Cd, or Hg are not yet understood. This would mean, however, that all these materials are metallic conductors, not semiconductors, as expected from the Zintl-Klemm concept. One might argue that the structures have both Cd/Ge positional disorder and defects on the 6*c* sites, *i.e.*, in analogy with Ba_8_Zn_*x*_Ge_46–*x*–*y*_☐_*y*_ and Ba_8_Cd_*x*_Ge_46–*x*–*y*_☐_*y*_ (☐ = vacancy) [18,19]. This certainly is a valid argument, which warrants further investigations. We will note here that, while for Ba_8_Zn_*x*_Ge_46–*x*–*y*_☐_*y*_ and Ba_8_Cd_*x*_Ge_46–*x*–*y*_☐_*y*_ the hypothesis for vacancies in the framework had been supported by the low occupation factors and elongated displacement parameters of the atoms neighboring the defects, none of these indicators of additional structural disorder are observed for the herein discussed structures. 

We also need to mention the fact that when checking the occupancies of the guest atoms in the reported structures **1** and **2**, they did not deviate from full. However, when the occupancies of the Cs sites in **3** were checked, it became obvious that site 2*a* is underoccupied (occupation factor 89.8(4)%). This is the center of the smaller pentagonal dodecahedral cage in the type-I clathrate, which might be not large enough to accommodate the very large Cs atom. Therefore, **3** is the only structure without 100% filled cages, refined as Cs_7.80(1)_Cd_3.65(6)_Ge_42.35_. To help the reader of this article appreciate the subtleties of the refinements Cs_7.80(1)_Cd_3.65(6)_Ge_42.35_ *vs.* Cs_8_Cd_3.65(6)_Ge_42.35_, the .CIF for the latter is provided as supporting evidence (please see the supplementary electronic information associated with this article). Despite the respectable residuals, quick convergence and quite reasonable anisotropic displacement parameters, the relatively deep hole in the difference electron density map (the Fourier synthesis indicates a hole of 4 *e^−^*/Å^3^ just 0.2 Å away from Cs2) is a telling sign that the respective position is not fully occupied. Indeed, when the occupation factor of Cs2 is freed, the difference Fourier map flattens out completely (Table 1 and Table 2).

**Figure 2:**
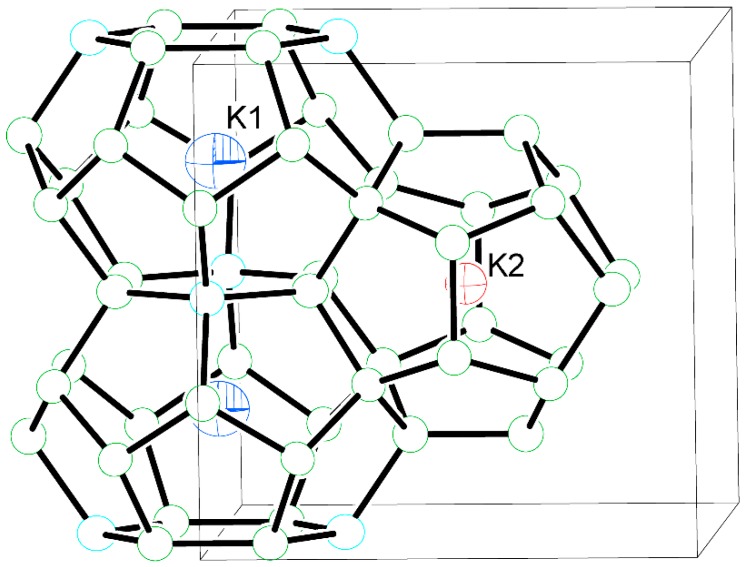
Representative fragment of the K_8_Cd_3.77(7)_Ge_42.23_ structure, drawn with thermal ellipsoids at the 98% probability level. U_*eq*_ (as one third of the trace of the orthogonalized U_*ij*_ tensor) for K1 is more than twice that of K2, hinting at small vibrations about the equilibrium position.

Lastly, while commenting on the size of the guest atoms and the clathrate cages, we should point the attention to the anisotropic displacement parameter of K1 in structure **1**, site 6*d* (Table 3). This atom is at the center of the larger tetrakaidecahedral cage (Figure 2). The size/shape of the thermal ellipsoid is not dependent on the site occupation factor (deviates from unity less than 3σ). The equivalent isotropic displacement parameter for K1 is, by far, the largest of all. Its slightly oblate shape may hint at off-centering (Table 3), but such positional disorder is very small and could not be resolved from the available crystallographic data. For comparison, the disorder in the type-I clathrates of tin, where the tetrakaidecahedral cages are even larger, is easily modeled from data collected on an in-house diffractometer [20,21,22]. Sr_8_Ga_16_Ge_30_ and Ba_8_Ga_16_Ge_30_ are other well-known examples of filler atoms being off-centered from their equilibrium positions, and both structures have been worked out from synchrotron and neutron diffraction data [23,24,25]. 

### 2.2. Structural Metrics

As seen in Table 1, the unit cell parameters of the three cubic type-I clathrates (space group *Pm* $\bar{3}n$ (No. 223)) increase from *a* = 10.8710(4) Å for K_8_Cd_3.77(7)_Ge_42.23_, to *a* = 10.9099(5) Å for Rb_8_Cd_3.65(7)_Ge_42.35_, to *a* = 10.9643(7) Å for Cs_7.80(1)_Cd_3.65(6)_Ge_42.35_. This increase can be attributed to the increase in size of the alkali metal atoms (*r*_K_ ≈ 2.03 Å, *r*_Rb_ ≈ 2.16 Å, *r*_Cs_ ≈ 2.35 Å, according to Pauling [26]). The small deviations in the refined Cd content must also have an effect on the unit cell volumes, but it is expected to be subtle (the difference in the refined Cd content is less than 2σ). Notice, however, that in the single-bonded Pauling radii for the Cd and Ge are quite different, *r*_Ge_ ≈ 1.24 Å, *r*_Cd_ ≈ 1.38 Å [26], and, as a result, the Cd substitutions to the Ge-based framework result in enlargement of the unit cell, clearly seen by comparing our data with the data for the known binary type-I clathrates K_8_Ge_44_☐_2_ [27] and Cs_8_Ge_44_☐_2_ [28], which have periodicity constants of *a* = 10.686(4) Å and *a* = 10.8238(2) Å, respectively. Of course, the presence of vacancies in the framework for K_8_Ge_44_☐_2_ and Cs_8_Ge_44_☐_2_ (☐ = vacancy) should not be overlooked, as it contributes to the contraction of the unit cell. Given that Cd and In are neighboring elements in the periodic chart with similar atomic radii (*r*_In_ ≈ 1.42 Å [26]), it is no surprise that the type-I clathrates from the systems K-In-Ge, Rb-In-Ge, and Cs-In-Ge show similar characteristics. The unit cell parameters of the latter increase are in the following order: *a* = 10.997(2) Å for K_8_In_8.14_Ge_37.86_; *a* = 11.033(2) Å for Rb_8_In_7.81_Ge_38.19_; *a* = 11.079(2) Å for Cs_8_In_8.22_Ge_37.78_ [29,30]. Another relevant comparison can be made between K_8_Cd_3.77(7)_Ge_42.23_ on the one hand, and K_8_Zn_4_Ge_42_ and K_8_Hg_3.19_Ge_42.81_ on the other [12,13]. The atomic size of Zn is slightly smaller than that of Cd (*r*_Zn_ ≈ 1.21 Å [26]), hence the smaller unit cell parameter for K_8_Zn_4_Ge_42_ (*a* = 10.7488(1) Å) [12]. Mercury and cadmium are very similar in size (*r*_Cd_ ≈ 1.38 Å; *r*_Hg_ ≈ 1.39 Å [26]), but the refined Hg content in K_8_Hg_3.19_Ge_42.81_ is lower that the Cd content in K_8_Cd_3.77(7)_Ge_42.23_, hence the smaller unit cell parameter for the former (*a* = 10.8489(13) Å) [13]. The same is true for Rb_8_Cd_3.65(7)_Ge_42.35_ (**2**) *vs.* Rb_8_Hg_3.03_Ge_42.97_ (*a* = 10.8750(13) Å [13]).

The net result of the substitution of the larger Cd for the smaller Ge is a small increase in the cage sizes, and therefore an expansion of the unit cells (*vide supra*). This can be also observed in the atomic distances (see Table 4), which fall in the intervals of *d*_Ge–Ge_ = 2.4931(4) to 2.52(1) Å for **1**, *d*_Ge–Ge_ = 2.5029(4) to 2.539(1) Å for **2**, and *d*_Ge–Ge_ = 2.5118(4) to 2.570(1) Å for **3**, respectively. Notice that, since Cd substitutes only at the 6*c* site, Cd-Cd contacts are avoided. This site only neighbors Ge1 and the corresponding *d*_Cd/Ge3-Ge1_ distances monotonically increase in the following order: 2.5858(6) Å for **1**, 2.5912(5) Å for **2**, and 2.6019(5) Å for **3**, respectively. The distances from the guest atoms in the cages to the framework follow the same trend (Table 4). 

## 3. Discussion

In 2014, we reported for the very first time the occurrence of the type-I and type-II clathrates Rb_5.20_Na_2.80(4)_Zn_3.85_Si_42.15(2)_ and Rb_8_Na_16_Zn_8.4_Si_127.6(1)_ [31]. Shortly after, another team reported the structure determination for K_8_Zn_3.46_Si_42.54_ and Rb_7.86_Zn_3.63_Si_42.37_ [14]. Subsequently, we set out to synthesize the Cd-analogs of these compounds, but these studies did not yield any clathrates.

As a result, we turned our attention to the germanium clathrates. We noticed that there were prior reports on K_8_Zn_4_Ge_42_ and the mixed-cation K_*x*_Ba_8–*x*_Zn_*y*_Ge_46–*y*_ [12]. K_8_Hg_3.19_Ge_42.81_ and Rb_8_Hg_3.03_Ge_42.97_ [13] have also been known for some time. The mixed-cation clathrates K_6_Eu_2_(Zn or Cd)_5_Ge_41_ also appear in the literature [11], but the corresponding crystallographic information is not available from the ICSD.

Given that mercury and cadmium are very similar in size, we thought it was surprising that the corresponding K-Cd-Ge, Rb-Cd-Ge, and Cs-Cd-Ge systems had not been carefully explored. This work fills this gap in the literature by detailing the structures of the type-I clathrates K_8_Cd_3.77(7)_Ge_42.23_, Rb_8_Cd_3.65(7)_Ge_42.35_, and Cs_7.80(1)_Cd_3.65(6)_Ge_42.35_. However, the latter clathrate phases are always co-crystallizing with small amounts of secondary phases, irrespective of the tried different synthesis routes. As mechanical separation was not achievable either, measurements of the transport properties could not be performed. 

In further investigations, we will aim to resolve the synthetic problems leading to impurity phases and study the transport properties. One of our more distant goals will be to explore the systems Cs-Na-Cd-Ge and Rb-Na-Cd-Ge. The mixed cations (large and small) can prove promising for synthesizing the hitherto unknown Cs_8_Na_16_Cd_12_Ge_124_ and Rb_8_Na_16_Cd_12_Ge_124_ type-II clathrates. We will recall that the crystal chemistry and the chemical bonding in many type-I clathrate structures are satisfactorily understood because they are more easily accessible in ternary systems. For type-II clathrates, the difficulty of synthesizing selectively new compounds remains an open challenge, which has been attributed to the fact that the cavities of the type-II clathrates have a larger difference in size. Therefore, we have speculated (and have shown experimentally on the examples of (Cs or Rb)_8_Na_16_(Si or Ge)_136_ [32]) that the directed synthesis of type-II can be greatly facilitated by choosing spatially different guest atoms. This idea, combined with the proper choice of a framework-substituent, can lead to new clathrate type-II, as demonstrated by the subsequent studies on Cs_8_Na_16_Ag_6.7_Ge_129.3_ [33] and Cs_8_Na_16_Cu_5_Ge_131_ [34]. Very recently, the applicability of notion that two types of different guest atoms will be preferred for the complete and ordered filling of both cavities in the type-II compounds was confirmed for the tin-based Cs_8_Ba_16_Ga_39.7_Sn_96.3_ and Rb_9.9_Ba_13.3_Ga_36.4_Sn_99.6_ [35], suggesting that the Cs-Ba-Cd-Ge and Rb-Ba-Cd-Ge systems are worthy candidates for further investigations.

## 4. Materials and Methods 

### 4.1. Synthesis

The syntheses were carried out by loading stoichiometric mixtures of the respective elements in suitably prepared Nb tubes, which were then sealed by arc-welding.

Due to the extremely high reactivity of the alkali metals to moisture and oxygen, all manipulations were done with great care in an argon-filled glove box. The atmosphere in the glovebox was maintained at O_2_/H_2_O level below 1 ppm. The elements were purchased from Alfa or Sigma-Aldrich with a stated purity higher than 99.9 wt % (metal basis). In order to carry out the reactions in a safe and reliable manner, the elements were weighed in a ratio of *A*:*M*:Ge = 8:4:42 (*A* = K, Rb, Cs). Total mass in each case was *ca.* 300 mg. After arc-welding, the Nb tubes with the reactants inside were enclosed in fused silica tubes. Under dynamic vacuum, the fused silica tubes were baked and then flame-sealed.

The samples were heated slowly in programmable muffle furnaces to 950 °C with a rate of 10 °C/h, annealed for 15 h, and then cooled down (rate −150 °C/h) to 650 °C, dwelled for 4 d, and cooled down (rate −5 °C/h) to room temperature. The obtained type-I clathrates were stable in air and moisture and were handled on the bench. Based on the powder X-ray diffraction patterns, the yields were high, estimated to be over 70–80 wt %, with some small impurity phases, yet unidentified, present in each specimen. 

### 4.2. Powder X-ray Diffraction

X-ray powder diffraction patterns of selected crystals were carried out at room temperature on a Rigaku MiniFlex powder diffractometer using Cu-Kα radiation. The data collection scans were done in θ-θ mode (2θ_max_ = 65°) with a step of 0.05° and 2 s/step counting time. The data were analyzed with the JADE 6.5 software package. The intensities and the positions of the experimentally observed peaks and those calculated based on the corresponding single-crystal structures matched very well with one another. 

### 4.3. Single-Crystal X-ray Diffraction

Single-crystal X-ray diffraction data were collected on a Bruker CCD-based diffractometer using graphite-monochromated Mo-Kα radiation (λ = 0.71073 Å). Temperature was maintained at 200(2) K. Suitable single-crystals from each compound were selected and cut to smaller dimensions (less than 0.1 mm) under mineral oil. The *SMART* [36] and *SAINTplus* [37] programs were used for the data collection, integration, and the global unit cell refinement from all data. Semi-empirical absorption correction based on equivalent reflections was applied with *SADABS* [38]. The structures were refined to convergence by full-matrix least-square methods on *F*^2^, as implemented in *SHELXTL* [39]. All sites were refined with anisotropic displacement parameters. 

Selected details of the data collections and structure refinement parameters are listed in Table 1. The atomic coordinates and equivalent isotropic displacement parameters are given in Table 2. The anisotropic displacement parameters are tabulated in Table 3, and selected interatomic distances are summarized in Table 4. Additional details of the crystal structure analyses may be requested from the Fachinformationszentrum Karlsruhe, D-76344 Eggenstein-Leopoldshafen (Karlsruhe, Germany) on quoting the depository numbers CSD-430998 for K_8_Cd_3.77(7)_Ge_42.23_, CSD-430999 for Rb_8_Cd_3.65(7)_Ge_42.35_, and CSD-431000 for Cs_7.80(1)_Cd_3.65(6)_Ge_42.35_, respectively.

### 4.4. Energy-Dispersive Analysis

Multiple crystals for each composition were analyzed by means of energy dispersive X-ray spectroscopy (EDX). Data were gathered using a JEOL 7400F electron microscope equipped with an INCA-OXFORD energy-dispersive spectrometer. Only the specified elements (*i.e*., no unrecognized impurities) could be detected in ratios consistent with the refined compositions.

## 5. Conclusions

With this paper, our team presented the synthesis and the structural characterization of the type-I clathrates K_8_Cd_3.77(7)_Ge_42.23_, Rb_8_Cd_3.65(7)_Ge_42.35_, and Cs_7.80(1)_Cd_3.65(6)_Ge_42.35_. In the future, we will be exploring the systems Cs-Na-Cd-Ge and Rb-Na-Cd-Ge. Based on the presented results and the overview of the current literature, we believe that using mixed cations (large and small) can yield the hitherto unknown Cs_8_Na_16_Cd_12_Ge_124_ and Rb_8_Na_16_Cd_12_Ge_124_ type-II clathrates.

## Figures and Tables

**Table 1:** Selected crystal data and structure refinement parameters for K_8_Cd_3.77(7)_Ge_42.23_ (**1**), Rb_8_Cd_3.65(7)_Ge_42.35_ (**2**), and Cs_7.80(1)_Cd_3.65(6)_Ge_42.35_ (**3**).

Compound	1	2	3
Fw/g·mol^−1^	3803.2	4170.2	4519.1
Crystal system	Cubic		
Space group	*Pm* $\bar{3}n$ (No. 223), *Z* = 1		
*a*/Å	10.8710(4)	10.9099(5)	10.9643(7)
*V*/Å^3^	1284.72(8)	1298.56(10)	1318.08(15)
*T*/K	200(2)		
Radiation	Mo Kα, λ = 0.71073 Å		
ρ/g·cm^−3^	4.92	5.33	5.69
μ/cm^−1^	264.3	329.7	304.7
data/restraints/parameters	291/0/17	318/0/17	327/0/18
*R*_1_ (*I* >2σ(*I*)) ^a^	0.0182	0.0178	0.0138
*wR*_2_ (*I* >2σ(*I*)) ^a^	0.0359	0.0365	0.0298
*R*_1_ (all data) ^a^	0.0241	0.0232	0.0180
*wR*_2_ (all data) ^a^	0.0378	0.0386	0.0314
GOF	1.156	1.064	1.116
largest peak & hole/e^−^·Å^−3^	0.54 & −0.57	0.62 & −0.85	0.63 & −0.56

^a^ R_1_ = ∑||F_o_| – |F_c_||/∑|F_o_|; *w*R_2_ = [∑[*w*(F_o_^2^ – F_c_^2^)^2^]/∑[*w*(F_o_^2^)^2^]]^1/2^, where *w* = 1/[σ^2^F_o_^2^ + (A·P)^2^ + (B·P)], and P = (F_o_^2^ + 2F_c_^2^)/3, A and B weight coefficients.

**Table 2:** Atomic coordinates and equivalent isotropic displacement parameters (U_*eq*_/Å^2^) for K_8_Cd_3.77(7)_Ge_42.23_ (**1**), Rb_8_Cd_3.65(7)_Ge_42.35_ (**2**), and Cs_7.80(1)_Cd_3.65(6)_Ge_42.35_ (**3**).

Atom	Site	*x*/*a*	*y*/*b*	*z*/*c*	Occupancy	U_*eq*_^a^
K_8_Cd_3.77(7)_Ge_42.23_						
K1	6*d*	0	^1^/_4_	^1^/_2_	100%	0.0358(7)
K2	2*a*	0	0	0	100%	0.0175(9)
Ge1	24*k*	0	0.30355(5)	0.11589(5)	100%	0.0129(2)
Ge2	16*i*	0.18327(4)	*x*	*x*	100%	0.0119(2)
Ge/Cd3	6*c*	^1^/_4_	0	^1^/_2_	37(1)/63(1)%	0.0137(3)
Rb_8_Cd_3.65(7)_Ge_42.35_						
Rb1	6*d*	0	^1^/_4_	^1^/_2_	100%	0.0254(3)
Rb2	2*a*	0	0	0	100%	0.0124(3)
Ge1	24*k*	0	0.30365(5)	0.11637(5)	100%	0.0123(2)
Ge2	16*i*	0.18356(3)	*x*	*x*	100%	0.0114(2)
Ge/Cd3	6*c*	^1^/_4_	0	^1^/_2_	39(1)/61(1)%	0.0127(3)
Cs_7.80(1)_Cd_3.65(6)_Ge_42.35_						
Cs1	6*d*	0	^1^/_4_	^1^/_2_	100%	0.0204(2)
Cs2	2*a*	0	0	0	89.8(4)%	0.0117(3)
Ge1	24*k*	0	0.30334(4)	0.11719(4)	100%	0.0123(1)
Ge2	16*i*	0.18370 (3)	*x*	*x*	100%	0.0116(2)
Ge/Cd3	6*c*	^1^/_4_	0	^1^/_2_	39(1)/61(1)%	0.0136(3)

^a^ U_*eq*_ is defined as one third of the trace of the orthogonalized U_*ij*_ tensor.

**Table 3:** Anisotropic displacement parameters (U_*ij*_/Å^2^) for K_8_Cd_3.77(7)_Ge_42.23_ (**1**), Rb_8_Cd_3.65(7)_Ge_42.35_ (**2**), and Cs_7.80(1)_Cd_3.65(6)_Ge_42.35_ (**3**).

Atom	U*_11_*	U*_22_*	U*_33_*	U*_23_*	U*_13_*	U*_12_*
K_8_Cd_3.77(7)_Ge_42.23_						
K1	0.038(1)	0.032(2)	=U*_11_*	0	0	0
K2	0.0175(9)	=U*_11_*	=U*_11_*	0	0	0
Ge1	0.0121(3)	0.0137(3)	0.0130(3)	0.0000(2)	0	0
Ge2	0.0119(2)	=U*_11_*	=U*_11_*	−0.0006(1)	=U*_23_*	=U*_23_*
Ge/Cd3	0.0157(5)	0.0127(4)	=U*_22_*	0	0	0
Rb_8_Cd_3.65(7)_Ge_42.35_						
Rb1	0.0278(4)	0.0205(6)	=U*_11_*	0	0	0
Rb2	0.0124(3)	=U*_11_*	=U*_11_*	0	0	0
Ge1	0.0119(3)	0.0127(3)	0.0124(3)	0.0003(2)	0	0
Ge2	0.0114(2)	=U*_11_*	=U*_11_*	−0.0005(1)	=U*_23_*	=U*_23_*
Ge/Cd3	0.0144(5)	0.0119 (3)	=U*_22_*	0	0	0
Cs_7.80(1)_Cd_3.65(6)_Ge_42.35_						
Cs1	0.0226(2)	0.0159(3)	=U*_11_*	0	0	0
Cs2	0.0117(3)	=U*_11_*	=U*_11_*	0	0	0
Ge1	0.0115(2)	0.0122(3)	0.0132(3)	0.0003(2)	0	0
Ge2	0.0116(2)	=U*_11_*	=U*_11_*	−0.0002(1)	=U*_23_*	=U*_23_*
Ge/Cd3	0.0158(4)	0.0125(3)	=U*_22_*	0	0	0

**Table 4:** Selected interatomic distances for K_8_Cd_3.77(7)_Ge_42.23_ (**1**), Rb_8_Cd_3.65(7)_Ge_42.35_ (**2**), and Cs_7.80(1)_Cd_3.65(6)_Ge_42.35_ (**3**).

Compound 1	*d*/Å	Compound 2	*d*/Å	Compound 3	*d*/Å
Ge1-Ge2 (2×)	2.4931(4)	Ge1-Ge2 (2×)	2.5029(4)	Ge1-Ge2 (2×)	2.5118(4)
Ge1-Ge1	2.520(1)	Ge1-Ge1	2.539(1)	Ge1-Ge1	2.570(1)
Ge1-Ge/Cd3	2.5858(6)	Ge1-Ge/Cd3	2.5912(5)	Ge1-Ge/Cd3	2.6019(5)
Ge2-Ge2	2.513(1)	Ge2-Ge2	2.511(1)	Ge2-Ge2	2.518(1)
Ge2-Ge1 (3×)	2.4931(4)	Ge2-Ge1 (3×)	2.5029(4)	Ge2-Ge1 (3×)	2.5118(4)
Ge/Cd3-Ge1 (4×)	2.5858(6)	Ge/Cd3-Ge1 (4×)	2.5912(5)	Ge/Cd3-Ge1 (4×)	2.6019(5)
K1-Ge1 (8×)	3.6789(4)	Rb1-Ge1 (8×)	3.6932(4)	Cs1-Ge1 (8×)	3.7167(4)
K1-Ge2 (8×)	4.0438(3)	Rb1-Ge2 (8×)	4.0564(3)	Cs1-Ge2 (8×)	4.0758(3)
K1-Ge/Cd3 (4×)	3.8435(2)	Rb1-Ge/Cd3 (4×)	3.8572(2)	Cs1-Ge/Cd3 (4×)	3.8765(2)
K1-Ge1 (4×)	4.2157(6)	Rb1-Ge1 (4×)	4.2262(5)	Cs1-Ge1 (4×)	4.2378(5)
K2-Ge2 (8×)	3.4507(7)	Rb2-Ge2 (8×)	3.4686(6)	Cs2-Ge2 (8×)	3.4885(6)
K2-Ge1 (12×)	3.5322(6)	Rb2-Ge1 (12×)	3.5477(5)	Cs2-Ge1 (12×)	3.5654(5)

## Supplementary Materials

The following are available online at www.mdpi.com/1996-1944/9/4/236/s1. Structure refinement for Cs_8_Cd_3.65(6)_Ge_42.35_, in .CIF.

## Abbreviations

The following abbreviations are used in this manuscript:

PGEC: Phonon-Glass Electron-Crystal
*Tt*: Tetrel, group 14 elements Si, Ge and Sn
*A*: group 1, 2 and 3 elements, which partially or fully occupy the cages within the clathrate framework
*M*: group 13 and 12 elements, as well as late transition metals, substituting framework atoms
☐: vacancy
